# Supplementary material for: NOS2 and COX2 Provide Key Spatial Targets that Determine Outcome in ER− Breast Cancer
Source: bioRxiv. 2023 Dec 23:2023.12.21.572859. Preprint. [Version 1] doi: 10.1101/2023.12.21.572859 (PMC10769386; doi:10.1101/2023.12.21.572859)
Supplement: Supplement 1 — Supplement Figure 1. Tumor analysis of NOS2 and COX2. A-B) Tumor NOS2 and COX2 expression at thresholds discerning strong, moderate, and weak signal intensities in Deceased and Alive patient tumors. C) Average cell intensity for NOS2 and COX2 was compared in Deceased and Alive patient tumors. * Mann Whitney p < 0.05. Supplement Figure 2. A) Upper panel showing quantification of immune and tumor (CK-SOX10) markers expressed in Deceased and Alive patient tumors. Lower panel INDO treatment upregulates mRNA expression of the thranscription factor IRF8, CLEC9a involved in anti-tumor immunity, chemokines CXCL9-11 that promote directional migration of immune cells, and IL27, which synergizes with IL12 to promote IFNγ production by CD4+, CD8+ T cells, and NKT cells. B) Comparison of whole tumor phenotypes in Deceased vs Alive patient tumors. C) Distribution plots of NOS2 vs CD8+ T cells of all samples showing distinct clustering of regional immune deserts, fully inflamed penetrating CD8+ T cells, and inflamed stroma restricted CD8+ T cells. D) Shows linear relationship between NOS2s and CD8 expression. Supplemental Figure 3. CD8+ T cell infiltration analysis in CD8+/−NOS2+/−COX2+/− phenotypes. A) Deceased tumor showing inflamed stromal restricted CD8+ T cells with limited tumor penetration at tumor-stroma interface in a whole tumor phenotype CD8+NOS2+COX2+. B) Deceased tumor showing an immune desert lacking CD8+ T cells in a whole tumor phenotype of CD8−NOS2+COX2+. C) Alive tumor showing fully inflamed CD8+ T cell penetration in phenotype of CD8+NOS2−COX2+/−. Supplement Figure 4. Regional distribution of phenotype of A) CD3CD8, B) NOS2 and COX2 in tumor and macrophage with <1% Macrophage-NOS2. [file media-1.pdf]

Comparison of NOS2 and COX2 Expression Signal Intensities

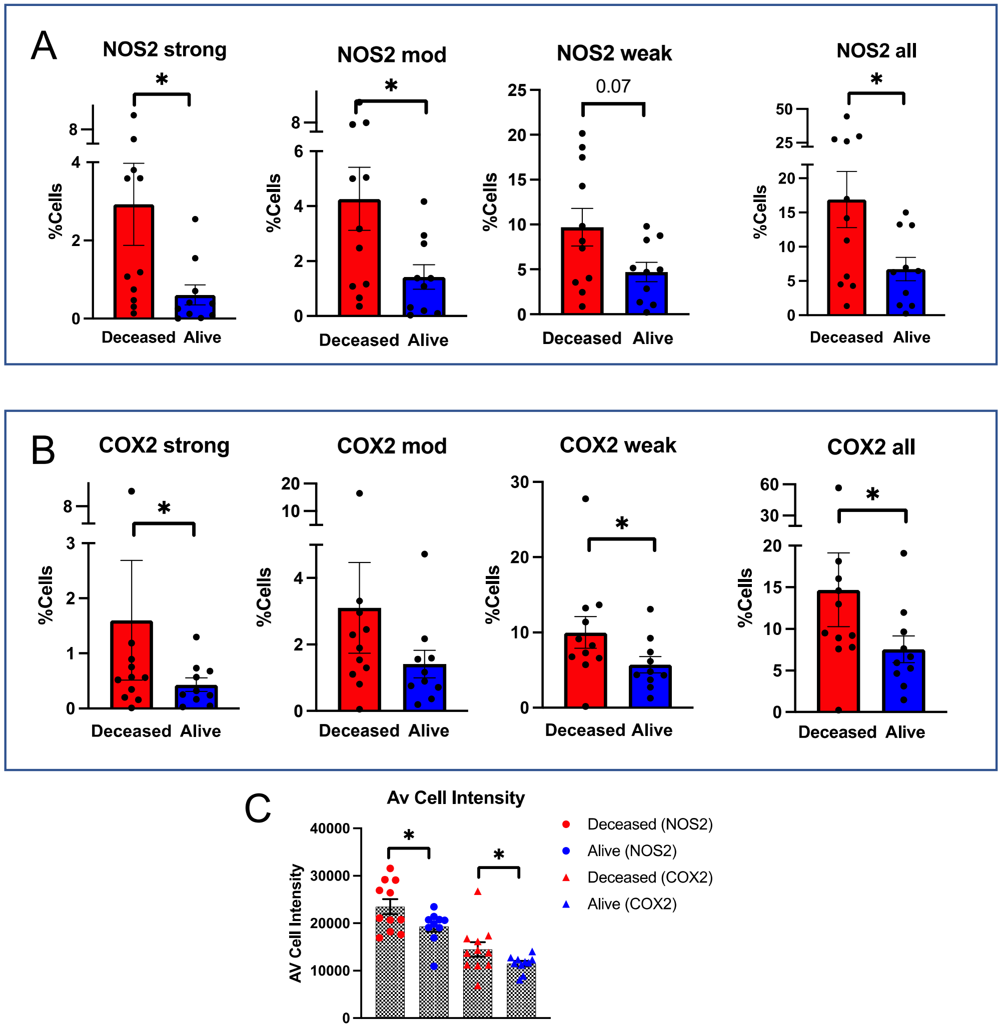

Suppl. Fig. 1

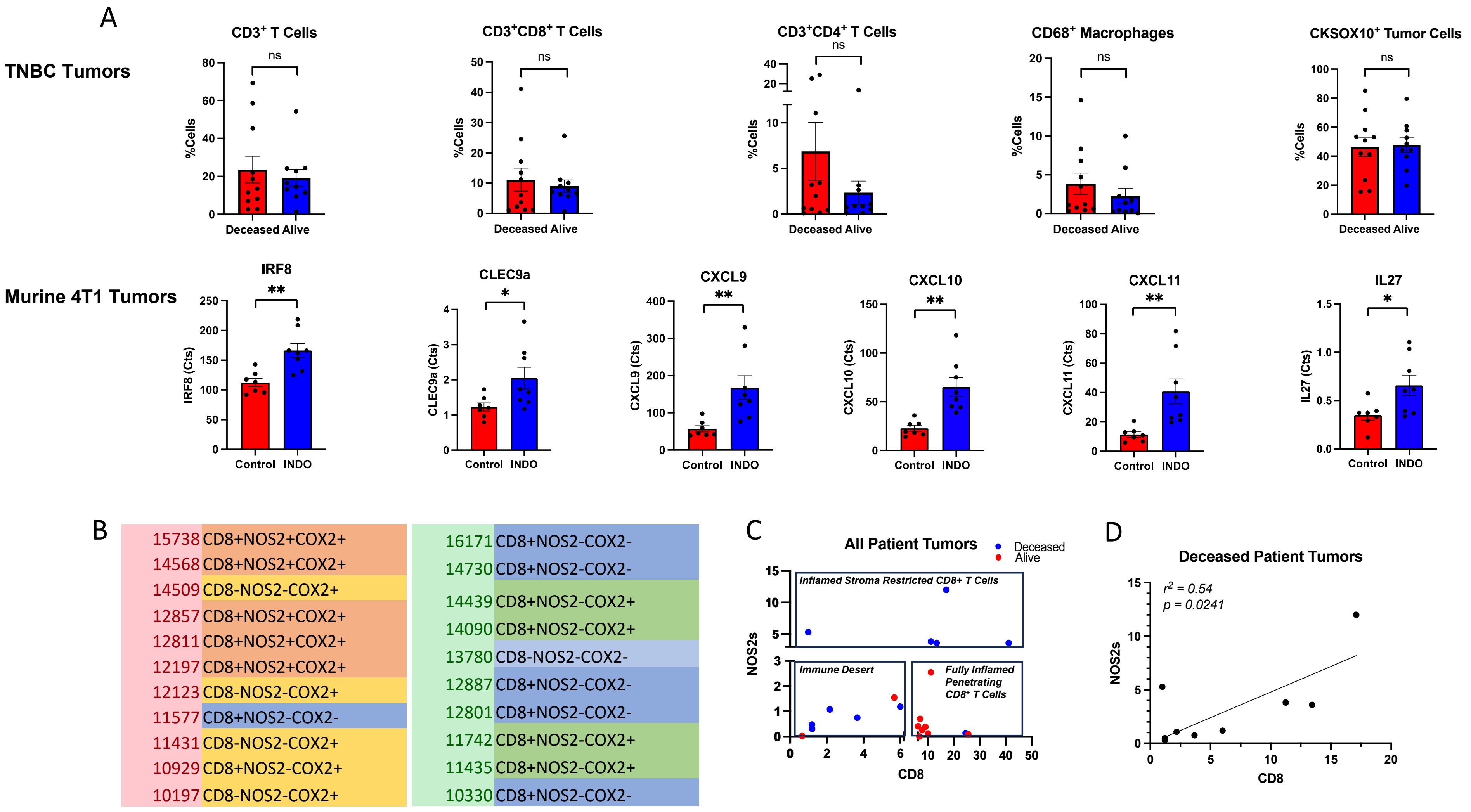
A
TNBC Tumors
CD3+ T Cells
CD3+CD8+ T Cells
CD3+CD4+ T Cells
CD68+ Macrophages
CKSOX10+ Tumor Cells
%Cells
ns
Deceased Alive
Murine 4T1 Tumors
IRF8
CLEC9a
CXCL9
CXCL10
CXCL11
IL27
IRF8 (Cts)
CLEC9a (Cts)
CXCL9 (Cts)
CXCL10 (Cts)
CXCL11 (Cts)
IL27 (Cts)
Control INDO
B
15738 CD8+NOS2+COX2+
14568 CD8+NOS2+COX2+
14509 CD8-NOS2-COX2+
12857 CD8+NOS2+COX2+
12811 CD8+NOS2+COX2+
12197 CD8+NOS2+COX2+
12123 CD8-NOS2-COX2+
11577 CD8+NOS2-COX2-
11431 CD8-NOS2-COX2+
10929 CD8+NOS2-COX2+
10197 CD8-NOS2-COX2+
16171 CD8+NOS2-COX2-
14730 CD8+NOS2-COX2-
14439 CD8+NOS2+COX2+
14090 CD8+NOS2+COX2+
13780 CD8-NOS2-COX2-
12887 CD8+NOS2-COX2-
12801 CD8+NOS2-COX2-
11742 CD8+NOS2-COX2+
11435 CD8+NOS2-COX2+
10330 CD8+NOS2-COX2-
C
All Patient Tumors
Deceased
Alive
Inflamed Stroma Restricted CD8+ T Cells
Immune Desert
Fully Inflamed Penetrating CD8+ T Cells
NOS2s
CD8
D
Deceased Patient Tumors
r2 = 0.54
p = 0.0241
NOS2s
CD8

Suppl. Fig. 2

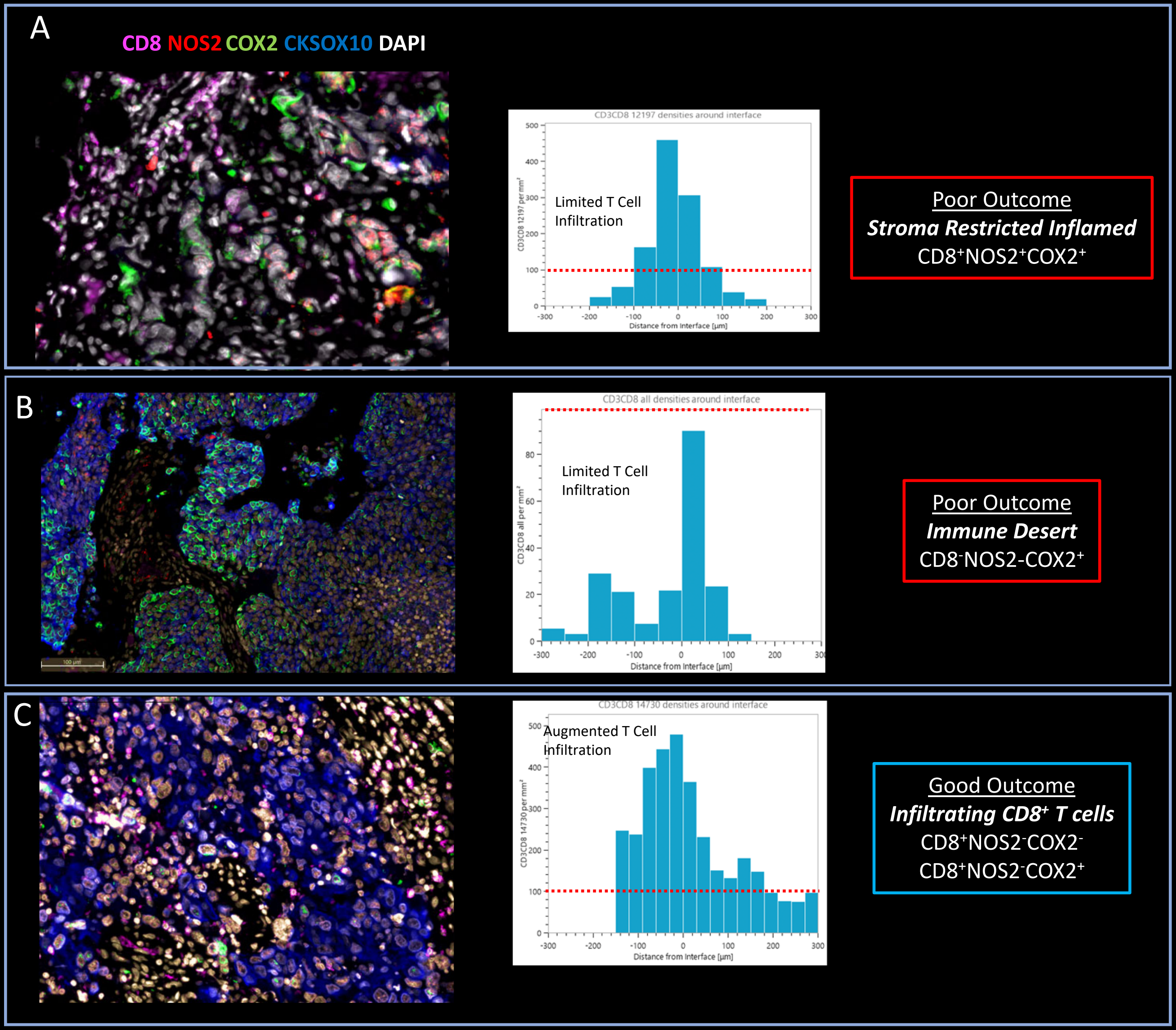
A
CD8 NOS2 COX2 CKSOX10 DAPI
CD3CD8 12197 densities around interface
Limited T Cell Infiltration
Distance from Interface [µm]
Poor Outcome
Stroma Restricted Inflamed
CD8$^+$NOS2$^+$COX2$^+$
B
CD3CD8 all densities around interface
Limited T Cell Infiltration
Distance from Interface [µm]
Poor Outcome
Immune Desert
CD8$^-$NOS2-COX2$^+$
C
CD3CD8 14730 densities around interface
Augmented T Cell Infiltration
Distance from Interface [µm]
Good Outcome
Infiltrating CD8$^+$ T cells
CD8$^+$NOS2$^-$COX2$^-$
CD8$^+$NOS2$^-$COX2$^+$

Suppl. Fig. 3

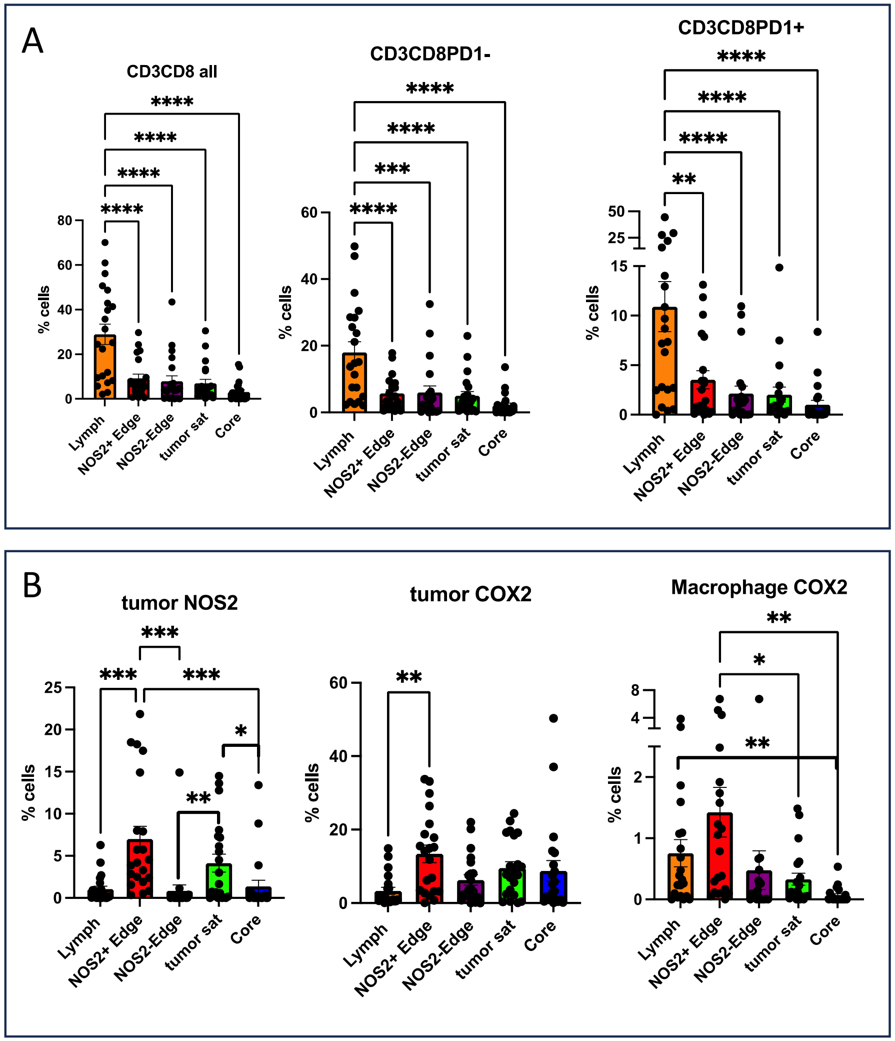
A
CD3CD8 all
CD3CD8PD1-
CD3CD8PD1+
% cells
Lymph
NOS2+ Edge
NOS2-Edge
tumor sat
Core
B
tumor NOS2
tumor COX2
Macrophage COX2

Suppl. Fig. 4
